# Urological Safety and COVID-19 Vaccinations

**DOI:** 10.3390/vaccines10111887

**Published:** 2022-11-08

**Authors:** Nazario Foschi, Paolo Emilio Santoro, Ivan Borrelli, Filippo Gavi, Carlotta Amantea, Pierluigi Russo, Umberto Moscato

**Affiliations:** 1 Department of Urology, Fondazione Policlinico Universitario Agostino Gemelli IRCCS, Largo Francesco Vito 1, 00168 Rome, Italy; 2 Department of Health Science and Public Health, Università Cattolica del Sacro Cuore, Largo Francesco Vito 1, 00168 Rome, Italy; 3 Department of Women, Children and Public Health Sciences, Fondazione Policlinico Universitario Agostino Gemelli IRCCS, Largo Francesco Vito 1, 00168 Rome, Italy; 4 Postgraduate School of Occupational Medicine, Università Cattolica del Sacro Cuore, Largo Francesco Vito 1, 00168 Rome, Italy

**Keywords:** COVID-19 vaccines, urology

## Abstract

Objective: To discuss the impact of COVID-19 vaccines on the urological field and to review the available data in the literature. Material and Methods: All the related reports and original articles discussing COVID-19 vaccines and their impact on the urological field were searched in PubMed, Scopus, and Web of Science. Results: There are few published articles discussing the COVID-19 vaccine impact on urology. Vaccine safety was confirmed in this field as no major side effects were described. AKI (Acute Kidney Injury) was reported in selected populations. However, about 1% of the side effects was urological. Rare genital complications, low urinary tract symptoms, and occasional gross hematuria were reported. Fertility seems to be not impaired after vaccination. A potential misinterpretation of radiological findings in the oncological field has been reported. Conclusions: In the literature, there are few studies regarding COVID-19 vaccines and their impact on the urological and andrological fields. We need more studies and extended follow-ups after repeated vaccinations in order to have more corroborating data particularly in selected populations, such as kidney transplant recipients and oncological patients.

## 1. Introduction

In March 2020, the World Health Organization (WHO) declared a pandemic. Currently, the SARS-CoV-2 virus has spread globally with several variants. The SARS-CoV-2 virus was arguably the greatest challenge in the modern era faced by healthcare systems all over the world. The COVID-19 pandemic has had a dramatic impact on urologists, urology residents, and urological patients [1]. Several hospitals in Spain collapsed due to the unbelievable numbers of patients with severe COVID-19 pneumonia causing the total cancellation of all activities in urology departments. In addition, a lot of resources and medical personnel were appointed to the care of COVID-19 patients, even urologists and urology residents. Whenever possible, outpatients visits were performed through online calls, and only cases considered urgent were attended, such as patients with high-risk cancers [2]. Moreover, residency training programs were also cancelled in several hospitals, including teaching activities resulting in a knowledge gap in terms of information and training [3]. Therefore, as pointed out by some studies in the literature, it was important for specialized medical schools to offer intelligent learning modalities for their residents to optimize virtual training in urology and other specialties [4,5]. During the COVID-19 pandemic, elective surgeries were suspended for many months, and only high-risk oncological cases were addressed, although an ambulatory follow-up and management of benign conditions, which can cause lower urinary tract symptoms (LUTS), were essential. In neurologic patients, for example, LUTS have a great impact on quality of life and social life, and their clinical conditions can seriously worsen if not properly addressed [6]. Frailty patients, such as oncological patients, patients with end-stage kidney disease, and kidney renal transplant recipients were at greater risk of developing severe COVID-19 symptoms and fatal complications, particularly in highly epidemic regions, and they were in need of special attention and precaution [7]. The higher risk of infection among the medical workforce facilitated healthcare worker burnout, and patients in some cases were left without proper care [8,9]. Because of the massive numbers of patients with COVID-19, many healthcare systems were on the brink of collapse. In this context, it is important to highlight again the usefulness of telemedicine and how certain clinical settings could be managed safely and effectively [10]. Greater use of telemedicine would have been of benefit, and such a system would have enabled continuity of care even at the most impactful times of COVID-19. Extreme measures were pursued in order to try to limit the virus diffusion and mortality, including extended periods of self-isolation and quarantine, use of personal protective equipment, travel limitations, and social distancing. In a critical situation like this, major efforts were undertaken globally to develop safe and effective vaccines quickly. In August 2021, the U.S. Food and Drug Administration finally approved the first COVID-19 vaccine [11].

Usually, the development of a vaccine is a lengthy process, taking seven to ten years, during which time research is conducted in successive stages that include quality testing, preclinical testing, and clinical trial phases in humans. In Europe, when a pharmaceutical company believes it can demonstrate the quality, safety, and efficacy of its product for a specific therapeutic indication, it submits a marketing authorization application to the European Medicine Agency (EMA). Only after approval by the EMA and authorization by the European Commission does the company start the large-scale production process. Vaccine studies against COVID-19 started in the spring of 2020, and in less than a year, the EMA recommended granting conditional marketing authorization to a first messenger RNA vaccine: mRNA (Comirnaty, BioNTech/Pfizer). Soon thereafter, on 6 January 2021, it granted a second one for the vaccine produced by Moderna. The development process has been accelerated at an unprecedented global level, yet no step in the process failed, thanks to the concurrence of several factors: earlier research on messenger RNA (mRNA) technology; studies on human coronaviruses related to SARS-CoV-2 (e.g., those that caused SARS—Severe acute respiratory syndrome—and MERS—Middle East respiratory syndrome); substantial human and economic resources made available within a tight timeframe, and parallel running of the various evaluation and study phases [12]. To contain the spread of COVID-19 and to reduce its severity, the WHO and governments from all around the world undertook unprecedented measures and promoted massive vaccination campaigns. As of 26 October 2022, a total of 12,830,378,906 vaccine doses had been administered [13]. COVID-19 vaccines were shown to be effective and to have few adverse events in the clinical trials [14,15]. Despite the strong data on the safety of the different types of COVID-19 vaccines, some people preferred not to be vaccinated fearing possible side effects, including among others, effects on fertility [16,17]. In the field of the Safety Platform for Emergency vACcines (SPEAC), the Brighton Collaboration Group developed a priority list of adverse events of special interest (AESI), with the aim of harmonizing vaccine safety evaluations for COVID-19. The SPEAC COVID-19 list of AESI was adopted by the WHO Global Advisory Committee on Vaccine Safety [18]. The main urological disorders included in the list are acute kidney injury, collapsed glomerulopathy, renal infarction, hypernatremia, ANCA-associated vasculitis with glomerulo-nephritis, and IgA vasculitis with nephritis; however, they refer to the COVID-19 disease and not to vaccination [18]. A tool for monitoring the safety of vaccines even after they have been approved and placed on the marketing market is pharmacovigilance and vaccinovigilance, which represent a complex set of activities aimed at continuously assessing all information on the safety of medicinal products and ensuring that the benefit/risk ratio remains favorable over time. The development of serious adverse events following the administration of the first dose of the vaccine is not an absolute contraindication to continuing the vaccination cycle. Each case is assessed by the vaccinating doctor, who decides on the patient’s suitability to receive the booster, based on the clinical, anamnestic, and pharmacological information available. No medicinal product can ever be considered risk-free. Each of us, when deciding to use a drug or receive a vaccination, should keep in mind that what he or she is doing is balancing the benefits with the risks. Verifying that the benefits of a vaccine outweigh the risks and minimizing these is the responsibility of the health authorities that regulate the marketing of medicinal products.

The continuous updating of the vaccine information sheets available and the redefinition of the indications of operability in the vaccination campaigns have contributed to fueling forms of unease and insecurity among healthcare workers during the COVID-19 vaccination campaign. In order to limit the medicolegal litigation on the matter, Law 76/2021 was enacted in Italy, which defines the exclusion of punishability for the vaccinating doctor, when, in the performance of correct vaccination procedures, the doctor has caused serious injury or death to the vaccinee as a result of the vaccination. It is assumed that the vaccinating physician, for the purposes of exclusion of punishability, followed the latest available guidelines, updated according to the vaccine datasheets, informed consent, and the guidelines published by the Ministry of Health (ministerial circulars) [19]. The law in question was created with the intention of protecting vaccinating doctors from possible medicolegal disputes due to possible vaccine-related side effects. Among the ten EU countries with the highest vaccination coverage against SARS-CoV-2, only Italy has adopted similar policies [20].

In the urological field, there is no clear and specific information on adverse effects from vaccination. This deficiency served as a motivation to perform a study, through a literature review, on the topic of vaccination safety in urology. Currently, few studies regarding vaccines and their impact on urological patients have been published, and reports on urological symptoms after COVID-19 vaccination are extremely rare. The aim of this study is to gather and resume the recommendations and data reported in the literature on the impact of COVID-19 vaccines on the urological field.

## 2. Materials and Methods

**Figure 1:**
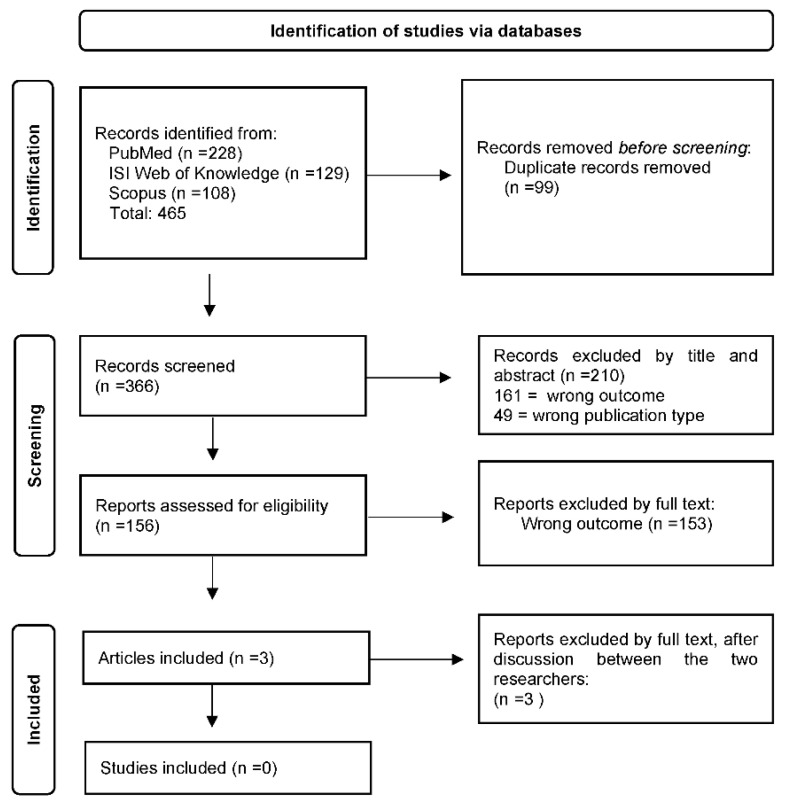
PRISMA flowchart. Symbols: n = number.

The present study, which was based on a review of the scientific literature and available documentation on vaccines found on the Web, was developed in two phases:

In the first phase, a review was attempted following the Preferred Reporting Items for Systematic Reviews and Meta-Analyses (PRISMA) Statements [21] through three databases: PubMed, Scopus, and Web of Science. Primary studies, in the English language, published between January 2020 and September 2022, were screened by using the following search terms: “Urological neoplasms”; “Urogenital disease”; “Kidney transplants”; “Urological disease”; “Fertility”; and “COVID-19 vaccination”. All the retrieved items were screened with the purpose to identify papers reporting connections between COVID-19 vaccines and urological symptoms and diseases. After retrieving the articles from all the selected databases, duplicate removal and the initial screening by title and abstract were performed through the website tool Rayyan [22], which allowed article screening by the researchers independently, following the double-blind methodology, in order to reduce selection bias (Figure 1). Only 3 articles were considered eligible for the aim of the review, although, upon reading the full text and after discussion between the two researchers, the articles concerned the COVID-19 disease and not specifically COVID-19 vaccination and urological diseases.

In the second phase of the research, due to the lack of original papers, the authors decided to include in this study data reported in case reports, systematic reviews, reviews, abstracts, and institutional sources.

## 3. Results

In the first phase of the study, the review resulted in 465 relevant articles across the three databases (PubMed, ISI Web of Knowledge, and Scopus). After removing duplicates, the initial search resulted in 366 eligible articles. Two researchers screened the articles blindly by title and abstract: 161 were excluded based on the wrong outcome; 49 articles were excluded according to the type of publication, and the remining 156 articles were screened by full text, and of these, only 3 articles were initially included in the revision and subsequently excluded, after discussion between the two researchers, as being not specifically relevant to the topic of vaccination (Figure 1). Any conflict about the inclusion or exclusion of the articles was resolved by internal discussion between the researchers.

In the second phase of the study, the authors consequently decided to extend the research to case reports, systematic reviews, reviews, abstracts, and institutional sources, which were divided in four major topics (andrology, oncology, kidney transplantation, and miscellaneous).

### 3.1. Andrology

The combination of COVID-19 vaccination and fertility was one of the major areas of research of great interest to the general population. Gonzalez et al. [17] conducted a study published in JAMA regarding the possible effects of the COVID-19 vaccines on fertility. Patients were prescreened in order to exclude possible fertility issues prior to vaccination. The exclusion criteria included a positive test result within 90 days. A semen sample was provided 2 to 7 days prior to receiving the first vaccine dose and approximately 2 months after the second dose. A sperm analysis before and after the two doses showed no significant decreases. This small cohort of 45 patients showed higher sperm parameters that can be considered as physiological and within normal ranges. The small number of patients, the short follow-up, and the lack of a control group were the limitations of this study. No differences in sperm motility, volume, or concentration were observed. It is essential to remember that a sperm analysis is an imperfect predictor of fertility potential. Overall, these preliminary results suggest no negative effects on the male reproductive system.

Winston et al. [23] wrote of a rare case of penile Mondor’s disease (PMD) 7 days after the first dose of a COVID-19 vaccine. A one-week treatment of acetaminophen and ibuprofen was sufficient to achieve a complete cure. PMD is an uncommon disease characterized by thrombosis of the superficial veins of the penis [24]. Several cases of PMD have been reported in patients infected with the SARS-CoV-2 virus [25,26]. It is important to mention that the AstraZeneca ChAdOx1-S vaccine can cause in rare cases a hypercoagulable state, and for that reason, in some countries, no further administration of this vaccine was provided [27,28].

### 3.2. Oncology

Hatakeyama S. et al. [29] presented a study at the Annual European Association of Urology Congress of 2022 in Amsterdam that evaluated the rates of an antispike antibody response to a BNT162b2 vaccine in patients with urological cancers. Data from 195 patients with prostate cancer (PC), 57 patients with urothelial cancer (UC), 28 patients with renal cell carcinoma (RCC), and 93 patients with kidney transplantation (KT) were retrospectively analyzed. Their results suggested that age (HR 0.95, *p* = 0.002), metastasis (HR0.25, *p* = 0.021), and immunosuppression (HR 0.003, *p* < 0.01) were significantly associated with seropositivity. Almost all the patients (90%) showed an adequate immunological response to the COVID-19 vaccine, although further research is needed to highlight the clinical implications of a lower antispike antibody response and their protective activity on a SARS-CoV-2 virus infection.

Recently, several cases of cancer patients with ^18^FDG PET CT evidence of metabolically active lymph nodes after COVID-19 vaccination have been reported [30,31]. PET CT is an important tool in the initial staging and follow-up of oncological patients and can dramatically change a patient’s treatment options and strategy. In a test such as PET CT, accuracy is essential [32]. Andresciani et al. [33] reported on a case of a male patient diagnosed with prostate cancer who underwent a PET CT after the COVID-19 vaccine. The PET CT scan showed a slight ^18^FDG uptake and enlargement of the left axillary lymph and paratracheal nodes. Because of the previous imaging reports, the patient’s clinical history, and the absence of a PSA increase, the patient underwent a new ^18^FDG PET-CT scan 14 weeks later showing an important decrease in the FCH uptake in all the previously involved regions. This case report highlights the importance on considering the vaccination history to evaluate imaging findings and to avoid false-positive reports and further unnecessary examinations.

### 3.3. Kidney Transplant

COVID-19 represents a great burden on kidney transplant recipients (KTRs). Daan et al. published a systematic review and meta-analysis [34] where they estimated that the risk of mortality is around 23% in KTRs regardless of comorbidities, sex, and age, highlighting the call to accelerate vaccination programs for KTRs. Some studies reported the serological response to COVID-19 in KTRs. Haskin et al. [35] reported a higher positive antibody response in adolescent and young adult KTRs than in adult KTRs. The antibody levels were lower in this group of patients than in patients with a previous COVID-19 infection. The majority of seronegative KTRs were previously treated with rituximab, and the time from the second vaccine dose to serologic testing was longer in seropositive than in seronegative patients [35]. During the follow-up, no vaccinated patients developed symptomatic COVID-19 disease.

KTRs are usually under immunosuppressive therapies for graft function. The American Society of Transplantation stated that the vaccine administration usually does not induce autoimmune reactions nor graft rejection rates [36,37].

COVID-19 vaccines have been administered in solid organ transplant recipients, and in their study, Ou et al. described a graft rejection only in 1 of 741 participants. Adverse symptoms were in line with expected vaccine reactogenicity, and severe side effects were rare [38]. Indeed, COVID-19 vaccination in these patients should be safe. KTRs should consider COVID-19 vaccination because they are at a high risk of suffering from severe COVID-19 symptoms. It is interesting that only 38% of KTRs had a humoral response after the COVID-19 vaccination; therefore, the dose of the SARS-CoV-2 vaccine needs to be augmented to maintain a sufficient humoral response [39].

### 3.4. Miscellaneous

Several cases of macroscopic hematuria were reported in patients with a history of biopsy-proven IgA nephropathy after the administration the COVID-19 vaccines [40,41,42]. Patients developed gross hematuria hours or days after the vaccine administration, and it generally resolved with supportive therapy only. Patients who developed AKI resolved it with steroid therapy. The authors suggested that a cell-mediated immune response was the cause of such a clinical presentation, but no further studies were performed. It is important to highlight that patients with kidney diseases are at an increased risk of mortality from severe COVID-19, and several studies have demonstrated the importance of COVID-19 vaccination in these patients, which is safe and effective [43,44]. Macroscopic hematuria can be the first symptoms of cancer in the kidney, urinary tract, or prostate.

Zhao et al. [45] queried the FDA Vaccine Adverse Event Reporting System (VAERS) for all the reported symptoms following the Pfizer-BioNTech and Moderna vaccines until February 2021. Out of 15,785 adverse events, about 1% included urologic symptoms. A total of 34 patients reported lower urinary tract symptoms, 14 patients reported hematuria; 41 patients reported urinary infection; 16 patients reported skin and or soft tissue issues; and 43 patients reported other non-specified urologic issues.

A case of genital necrosis with cutaneous thrombosis 26 days after the administration of the COVID-19 vaccine in an 84-year-old woman in Japan was reported. No further studies were perpetrated nor were other similar cases reported in the literature [46]. Acute genital ulceration was reported in women with COVID-19. It is a rare, nonsexually acquired condition characterized by the sudden onset of ulcerations of the vulva in young girls and women. Popatia et al. [47] reported acute genital ulcerations in a 12-year-old three days after receiving the second dose of the COVID-19 vaccine. Therapy consisted of local treatment with lidocaine and triamcinolone and symptomatic treatment. The genital ulcerations and her symptoms resolved in 10 days.

## 4. Conclusions

According to the literature review performed, there are few studies that examine the issue of COVID-19 vaccine-related urological damage or disorders directly; therefore, there is no current evidence to suggest that COVID-19 vaccines are associated with adverse urological outcomes. The SPEC (Safety Platform for Emergency vACcines) list of AESI (Adverse Event of Special Interest) also shows no specific urological complications caused by the vaccine. Therefore, considering the limited postauthorization evidence on urological outcomes, studies including these results are warranted in order to maintain confidence in the vaccine among urologists and their patients. We need more studies and extend follow-ups after repeated vaccinations in order to have more corroborating data particularly in selected populations, such as kidney transplant recipients and oncological patients.

## Data Availability

Not applicable.

## References

[1] Puliatti S., Eissa A., Eissa R., Amato M., Mazzone E., Dell’Oglio P., Sighinolfi M.C., Zoeir A., Micali S., Bianchi G. (2020). COVID-19 and urology: A comprehensive review of the literature: COVID-19 and urology. BJU Int..

[2] Hevia V., Lorca J., Hevia M., Domínguez A., López-Plaza J., Artiles A., Álvarez S., Sánchez Á., Fraile A., López-Fando L. (2020). Pandemia COVID-19: Impacto y reacción rápida de la Urología. Actas Urol. Esp..

[3] Chan M.C., Yeo S.E.K., Chong Y.L., Lee Y.M. (2020). Stepping Forward: Urologists’ Efforts During the COVID-19 Outbreak in Singapore. Eur Urol..

[4] Campi R., Amparore D., Checcucci E., Claps F., Teoh J.Y.C., Serni S., Scarpa R.M., Porpiglia F., Carrion D.M., Gomez Rivas J. (2021). Explorando la perspectiva de los residentes sobre las modalidades y contenidos de aprendizaje inteligente para la educación virtual de urología: Lección aprendida durante la pandemia de la COVID-19. Actas Urol. Esp..

[5] Claps F., Amparore D., Esperto F., Cacciamani G., Fiori C., Minervini A., Liguori G., Trombetta C., Porpiglia F., On Behalf of European Society of Residents in Urology (ESRU) (2020). Smart Learning for Urology Residents during the COVID-19 Pandemic and beyond: Insights from a Nationwide Survey in Italy. Minerva Urol. Nefrol..

[6] Bientinesi R., Coluzzi S., Gavi F., Nociti V., Gandi C., Marino F., Moretto S., Mirabella M., Bassi P., Sacco E. (2022). The Impact of Neurogenic Lower Urinary Tract Symptoms and Erectile Dysfunctions on Marital Relationship in Men with Multiple Sclerosis: A Single Cohort Study. J. Clin. Med..

[7] Gori A., Dondossola D., Antonelli B., Mangioni D., Alagna L., Reggiani P., Bandera A., Rossi G. (2020). Coronavirus disease 2019 and transplantation: A view from the inside. Am. J. Transpl..

[8] Adams J.G., Walls R.M. (2020). Supporting the Health Care Workforce During the COVID-19 Global Epidemic. JAMA.

[9] Antonucci M., Recupero S.M., Marzio V., De Dominicis M., Pinto F., Foschi N., Di Gianfrancesco L., Bassi P., Ragonese M. (2020). El impacto de la COVID-19 en las admisiones al servicio de urgencias, hospitalizaciones y manejo clínico de la urolitiasis en el centro de Italia: Análisis multicéntrico. Actas Urol. Esp..

[10] Novara G., Checcucci E., Crestani A., Abrate A., Esperto F., Pavan N., De Nunzio C., Galfano A., Giannarini G., Gregori A. (2020). Telehealth in Urology: A Systematic Review of the Literature. How Much Can Telemedicine Be Useful During and After the COVID-19 Pandemic?. Eur. Urol..

[11] Patel R., Kaki M., Potluri V.S., Kahar P., Khanna D. (2022). A comprehensive review of SARS-CoV-2 vaccines: Pfizer, Moderna & Johnson & Johnson. Hum. Vaccines Immunother..

[12] EpiCentro Sviluppo, Valutazione e Approvazione dei Vaccini Contro COVID-19. https://www.epicentro.iss.it/vaccini/covid-19-sviluppo-valutazione-approvazione.

[13] WHO Coronavirus (COVID-19) Dashboard. https://covid19.who.int.

[14] Munro A.P.S., Janani L., Cornelius V., Aley P.K., Babbage G., Baxter D., Bula M., Cathie K., Chatterjee K., Dodd K. (2021). Safety and immunogenicity of seven COVID-19 vaccines as a third dose (booster) following two doses of ChAdOx1 nCov-19 or BNT162b2 in the UK (COV-BOOST): A blinded, multicentre, randomised, controlled, phase 2 trial. Lancet.

[15] Heath P.T., Galiza E.P., Baxter D.N., Boffito M., Browne D., Burns F., Chadwick D.R., Clark R., Cosgrove C., Galloway J. (2021). Safety and Efficacy of NVX-CoV2373 COVID-19 Vaccine. N. Engl. J. Med..

[16] Szilagyi P.G., Thomas K., Shah M.D., Vizueta N., Cui Y., Vangala S., Kapteyn A. (2021). National Trends in the US Public’s Likelihood of Getting a COVID-19 Vaccine—April 1 to December 8, 2020. JAMA.

[17] Gonzalez D.C., Nassau D.E., Khodamoradi K., Ibrahim E., Blachman-Braun R., Ory J., Ramasamy R. (2021). Sperm Parameters Before and After COVID-19 mRNA Vaccination. JAMA.

[18] Law B., Matthew D. SO_2_-D2.1.1 Priority List of COVID-19 Adverse Events of Special Interest: Quarterly Update. https://brightoncollaboration.us/wp-content/uploads/2021/01/SO2_D2.1.1_V1.1_COVID-19_AESI-update-Sep2020.pdf.

[19] Beccia F., Amantea C., Rossi M.F., Daniele A., Santoro P.E., Borrelli I., Marazza M., Boccia S., Ricciardi W., Moscato U. (2021). Legal responsibility of vaccinating doctor. G. Ital. Med. Lav. Ergon..

[20] Amantea C., Rossi M.F., Santoro P.E., Beccia F., Gualano M.R., Borrelli I., Pinto da Costa J., Daniele A., Tumminello A., Boccia S. (2022). Medical Liability of the Vaccinating Doctor: Comparing Policies in European Union Countries during the COVID-19 Pandemic. Int. J. Environ. Res. Public Health.

[21] Shamseer L., Moher D., Clarke M., Ghersi D., Liberati A., Petticrew M., Shekelle P., Stewart L.A. (2015). Preferred reporting items for systematic review and meta-analysis protocols (PRISMA-P) 2015: Elaboration and explanation. BMJ.

[22] Ouzzani M., Hammady H., Fedorowicz Z., Elmagarmid A. (2016). Rayyan—A web and mobile app for systematic reviews. Syst. Rev..

[23] Winston J., Munien K. (2022). Superficial Thrombophlebitis of the Penis following AstraZeneca ChAdOx1-S Vaccination: A Rare Venous Thromboembolic Complication. Eur. J. Case Rep. Intern. Med..

[24] Öztürk H. (2014). Penile Mondor’s disease. Basic Clin. Androl..

[25] Eren M.T., Özveri H., Kurtoğlu H. (2021). Penile Mondor’s in a COVID-19 patient on prophylactic anti-thrombosis with rivaroxaban: A case report. Afr. J. Urol..

[26] Lessiani G., Boccatonda A., D’Ardes D., Cocco G., di Marco G., Schiavone C. (2020). Mondor’s Disease in SARS-CoV-2 Infection: A Case of Superficial Vein Thrombosis in the Era of COVID-19. Eur. J. Case Rep. Intern. Med..

[27] Pottegård A., Lund L.C., Karlstad Ø., Dahl J., Andersen M., Hallas J., Lidegaard Ø., Tapia G., Gulseth H.L., Ruiz P.L. (2021). Arterial events, venous thromboembolism, thrombocytopenia, and bleeding after vaccination with Oxford-AstraZeneca ChAdOx1-S in Denmark and Norway: Population based cohort study. BMJ.

[28] Ramessur R., Saffar N., Czako B., Agarwal A., Batta K. (2021). Cutaneous thrombosis associated with skin necrosis following Oxford-AstraZeneca COVID-19 vaccination. Clin. Exp. Dermatol..

[29] Hatakeyama S., Yoneyama T., Hamaya T., Togashi K., Narita T., Fujita N., Yamamoto H., Yoneyama T., Hashimoto Y., Ohyama C. (2022). Antibody responses to BNT162b2 mRNA COVID-19 vaccine in healthcare workers and patients with urological diseases in Japan. Eur. Urol..

[30] Nawwar A.A., Searle J., Hopkins R., Lyburn I.D. (2021). False-Positive Axillary Lymph Nodes on FDG PET/CT Resulting From COVID-19 Immunization. Clin. Nucl. Med..

[31] Özütemiz C., Krystosek L.A., Church A.L., Chauhan A., Ellermann J.M., Domingo-Musibay E., Steinberger D. (2021). Lymphadenopathy in COVID-19 Vaccine Recipients: Diagnostic Dilemma in Oncologic Patients. Radiology.

[32] Fortuin A., de Rooij M., Zamecnik P., Haberkorn U., Barentsz J. (2013). Molecular and Functional Imaging for Detection of Lymph Node Metastases in Prostate Cancer. Int. J. Mol. Sci..

[33] Andresciani F., Ricci M., Grasso R.F., Zobel B.B., Quattrocchi C.C. (2022). COVID-19 vaccination simulating lymph node progression in a patient with prostate cancer. Radiol. Case Rep..

[34] Kremer D., Pieters T.T., Verhaar M.C., Berger S.P., Bakker S.J.L., Zuilen A.D., Joles J.A., Vernooij R.W.M., van Balkom B.W.M. (2021). A systematic review and meta-analysis of COVID-19 in kidney transplant recipients: Lessons to be learned. Am. J. Transpl..

[35] Haskin O., Ashkenazi-Hoffnung L., Ziv N., Borovitz Y., Dagan A., Levi S., Koren G., Hamdani G., Levi-Erez D., Landau D. (2021). Serological Response to the BNT162b2 COVID-19 mRNA Vaccine in Adolescent and Young Adult Kidney Transplant Recipients. Transplantation.

[36] Cordero E., Bulnes-Ramos A., Aguilar-Guisado M., González Escribano F., Olivas I., Torre-Cisneros J., Gavaldá J., Aydillo T., Moreno A., Montejo M. (2020). Effect of Influenza Vaccination Inducing Antibody Mediated Rejection in Solid Organ Transplant Recipients. Front. Immunol..

[37] Baluch A., Humar A., Eurich D., Egli A., Liacini A., Hoschler K., Campbell P., Berka N., Urschel S., Wilson L. (2013). Randomized Controlled Trial of High-Dose Intradermal Versus Standard-Dose Intramuscular Influenza Vaccine in Organ Transplant Recipients: Intradermal Vaccine in Transplant. Am. J. Transpl..

[38] Ou M.T., Boyarsky B.J., Motter J.D., Greenberg R.S., Teles A.T., Ruddy J.A., Krach M.R., Jain V.S., Werbel W.A., Avery R.K. (2021). Safety and Reactogenicity of 2 Doses of SARS-CoV-2 Vaccination in Solid Organ Transplant Recipients. Transplantation.

[39] Kamar N., Abravanel F., Marion O., Couat C., Izopet J., Del Bello A. (2021). Three Doses of an mRNA Covid-19 Vaccine in Solid-Organ Transplant Recipients. N. Engl. J. Med..

[40] Plasse R., Nee R., Gao S., Olson S. (2021). Acute kidney injury with gross hematuria and IgA nephropathy after COVID-19 vaccination. Kidney Int..

[41] Rahim S.E.G., Lin J.T., Wang J.C. (2021). A case of gross hematuria and IgA nephropathy flare-up following SARS-CoV-2 vaccination. Kidney Int..

[42] Perrin P., Bassand X., Benotmane I., Bouvier N. (2021). Gross hematuria following SARS-CoV-2 vaccination in patients with IgA nephropathy. Kidney Int..

[43] Rubin E.J., Baden L.R., Morrissey S. (2020). Audio Interview: A Look at Covid-19 Prevention and Care in 2020. N. Engl. J. Med..

[44] Windpessl M., Bruchfeld A., Anders H.J., Kramer H., Waldman M., Renia L., Ng L.F.P., Xing Z., Kronbichler A. (2021). COVID-19 vaccines and kidney disease. Nat. Rev. Nephrol..

[45] Zhao H., Souders C., Carmel M., Anger J.T. (2021). Low Rates of Urologic Side Effects Following Coronavirus Disease Vaccination: An Analysis of the Food and Drug Administration Vaccine Adverse Event Reporting System. Urology.

[46] Kuzumi A., Yoshizaki A., Chiba K., Mitsuo S., Matsuda K.M., Norimatsu Y., Nagai K., Omatsu J., Miyake T., Sato S. (2022). Genital necrosis with cutaneous thrombosis after COVID-19 mRNA vaccination. J. Eur. Acad. Dermatol. Venereol..

[47] Popatia S., Chiu Y.E. (2022). Vulvar aphthous ulcer after COVID-19 vaccination. Pediatr. Dermatol..

